# Socioeconomic status and COVID‐19‐related cases and fatalities in the world: A cross‐sectional ecological study

**DOI:** 10.1002/hsr2.628

**Published:** 2022-05-05

**Authors:** Ahmad Faramarzi, Javad Javan‐Noughabi, Sayed Ali Mousavi, Farshad Bahrami Asl, Hamidreza Shabanikiya

**Affiliations:** ^1^ Department of Health Management and Economics, School of Public Health Urmia University of Medical Sciences Urmia Iran; ^2^ Social Determinants of Health Research Center Mashhad University of Medical Sciences Mashhad Iran; ^3^ Department of Health Economics and Management Sciences, School of Health Mashhad University of Medical Sciences Mashhad Iran; ^4^ Department of Health Shoushtar Faculty of Medical Science Shoushtar Iran; ^5^ Environmental Health Engineering Department, School of Health Urmia University of Medical Sciences Urmia Iran

**Keywords:** COVID‐19, mortality, regression, socioeconomic

## Abstract

**Background and Aims:**

The COVID‐19 pandemic poses an extraordinary threat to global public health. We designed an ecological study to explore the association between socioeconomic factors and the COVID‐19 outcomes in 184 countries, using the geographic map and multilevel regression models.

**Methods:**

We conducted a cross‐sectional ecological study in 184 countries. We performed regression analysis to assess the association of various socioeconomic variables with COVID‐19 outcomes in 184 countries, using ordinary least squares and multilevel modeling analysis. We performed two‐level analyses with countries at Level 1 and geographical regions at Level 2 in multilevel modeling analysis, using the same set of predictor variables used in ordinary least squares.

**Results:**

There was a significant relationship between COVID‐19 cases rate (Log) per 100,000 inhabitants‐day at risk with human development index (HDI), percentage of the urban population, unemployment, and cardiovascular disease prevalence. The results displayed that the variances are varied between Level 1 (country level) and Level 2 (World Health Organization [WHO] regions), meaning that the geographic distribution represented a proportion of the changes in the COVID‐19 outcomes.

**Conclusion:**

The study suggests that in addition to the socioeconomic status affects the COVID‐19 outcomes, countries' geographical location makes a part of changes in outcomes of diseases. Therefore, health policy‐makers could overcome morbidity and mortality in COVID‐19 by controlling the socioeconomics factors.

## 1 INTRODUCTION

In December 2019, a new severe acute respiratory syndrome coronavirus was reported in Wuhan, China. While the World Health Organization (WHO) is named the novel coronavirus disease (COVID‐19).^1^, ^2^ The widespread transmission of COVID‐19, which has spread in more than 188 counties until July 20, 2020, led to its being identified as a pandemic.^3^, ^4^ Up to May 26, 2021, according to a report by WHO, there were 167.01 million cases, and 3.47 million deaths were reported.^5^

Due to the high rate of transmission by COVID‐19, it is essential to identify the influential factors for the morbidity and mortality of the disease. Studies have shown that individual factors such as age, sex, and underlying diseases, including diabetes, hypertension, cardiovascular disease, chronic obstructive pulmonary disease (COPD), kidney failure, and cancer, are significant predictors for COVID‐19‐related outcomes, that is, incidence, mortality, and fatality.^6^, ^7^, ^8^, ^9^ In this regard, Yang et al.^10^ conducted a meta‐analysis, including eight studies with 46,248 COVID‐19 patients. They indicated that the most prevalent comorbidities were hypertension (17%), diabetes (8%), cardiovascular disease (5%), and respiratory system disease (2%). Also, the results revealed that the odds ratio of hypertension, respiratory system disease, cardiovascular disease in severe patients was 2.36, 2.46, and 3.42, respectively, compared to nonsevere patients.

Besides, some socioeconomics and environmental factors might play a facilitating role in populations' susceptibility and vulnerability. Studies have shown a significant relationship between COVID‐19 and socioeconomics variables such as public transportation per capita, aging index, poverty index, employment rate, gross domestic product (GDP) per capita, and the workforce employed in essential services.^11^, ^12^, ^13^, ^14^ An analysis of 3.99 million individuals with COVID‐19 in Brazil revealed a significant association between social and income inequalities and the COVID‐19 mortality rate.^15^ Wildman reported that a 1% increase in the Gini coefficient in the OECD countries is associated with an approximately 4% and 5% increase in incidence and mortality rate per million, respectively.^14^

Although, there were some studies related to the role of socioeconomic status and demographic factors on the COVID‐19‐related cases and fatalities. However, these studies were only conducted in a particular country or region, the data were obtained during the early phase of the pandemic, and disease was not reported in many countries. Therefore, this ecological study aimed to explore the association between socioeconomic factors and the COVID‐19 outcomes in 184 countries, using the geographic map and multilevel regression models.

## 2 MATERIALS AND METHODS

### 2.1 Study population

We conducted a cross‐sectional ecological study in 184 countries around the globe. We selected the countries based on the following criteria; first, up to February 12, 2021, at least one confirmed case of COVID‐19 has been reported. Second, the data on COVID‐19 and socioeconomic variables were available to them.

### 2.2 Variables and data sources

We considered the data related to COVID‐19 as outcome variables and socioeconomic factors as independent variables. The daily data on recorded cases and deaths by COVID‐19 are summed up to February 12, 2021 from WHO reports for selected countries.^5^ We estimated population‐time at risk as a product of the total population multiplied by the number of days since the first symptom for the first confirmed case at each country. Then, the cumulative incidence and mortality rate was calculated using the population‐time at risk as a denominator. We also computed the case fatality rate by dividing the number of COVID‐19 deaths by the confirmed cases.

We retrieved the data regarding socioeconomic variables such as human development index (HDI), total population, women population (% total population), the population aged over 65 years (% total population), the population aged over 75 years (% total population), population density (number per km^2^), urban population (% total population), median age (year), total unemployment rate (%), years of schooling (years), and education index from reports of United Nations Development Program (UNDP). The education index ran from zero to one, an average of mean years of schooling (of adults) and expected years of schooling (of children). The data on the GDP per capita and health expenditure per capita was collected from World Bank data.

We obtained data about the prevalence of cardiovascular, diabetes and kidney, and chronic obstructive pulmonary disease (COPD) per 100,000 population using the global burden disease study in 2019.

### 2.3 Statistical analysis

We first described the COVID‐19 incidence and mortality rates, using the mean, standard deviation, median, and interquartile range (IQR) per 100,000 population‐time at risk. The fatality rate was also reported as the percentage. Besides, the socioeconomic determinants and disease burden variables such as cardiovascular, diabetes and kidney, COPD were reported. We depicted the distribution of COVID‐19‐related variables by employing the shapefiles of countries worldwide.

We used Pearson's and Spearman's correlation coefficients to quantify the strengths of associations between morbidity, mortality, and case fatality rate with covariate variables. The outcome variables, that is, morbidity, mortality, and fatality rate, were transformed into the common logarithm (log_10_) to adjust for the normal distribution. The death number was zero in 10 countries because zero cannot be transformed to the common logarithm, so we added 0.00001 per inhabitants‐day at risk to the mortality rate. A two‐sided *t*‐test was applied to evaluate significant differences between COVID‐19 outcomes and socioeconomic characteristics.

We performed regression analysis to assess the association of various socioeconomic variables with COVID‐19 outcomes in 184 countries. Variables were selected as independent variables with a correlation coefficient greater than 0.5. We first used the ordinary least squares regression model as follows:

$$\mathrm{Log}\left({\text{COVID}\_\text{outcome}}_{i}\right){=\alpha +\beta }_{1}{\text{HDI}+\beta }_{2}{\text{URP}+\beta }_{3}{\text{UNE}+\beta }_{4}{\text{CRD}+\beta }_{5}{\text{DK}+\beta }_{6}{\text{POP}+u}_{i},$$

"Wher Log (COVID_outcome_*i*_) represents either cases or mortality rate per 100,000 person‐day at risk for country *i*; HDI is the human development index which is divided into four groups: low HDI, medium HDI, high, and very high HDI, according to the UNDP; URP and POP donate the urban and elderly population as a percentage of the total population, respectively; UNE is total unemployment rate; CRD and DK are the prevalence of cardiovascular, diabetes, and kidney per 100,000 population; *β* is the vector of regression coefficients; and *u_i_* is the error term.

Then, we applied multilevel modeling for some plausible reasons. First, we assessed the impact of geographical environment on COVID‐19 outcomes, 184 countries classified into six groups based on WHO regions, including Western Pacific, Eastern Mediterranean, European, South‐East Asia, Pan American, and African region. Second, some of the data had a nested structure. There is usually collinearity in data with a hierarchical structure, so we applied the mixed model analysis to avoid this problem. Third, we performed a sensitivity analysis, running multilevel modeling analysis to examine whether the effects of socioeconomic variables on the COVID‐19 variables were adjusted.

In multilevel analysis, we performed two‐level analyses with countries at Level 1 and geographical regions at Level 2, using the same set of predictor variables used in ordinary least squares. The multilevel regression equation used is as the following formula:

$$\mathrm{Log}\left({\text{COVID}\_\text{outcome}}_{\text{ij}}\right){\;=\alpha }_{0}{+\;\beta }_{1}{\text{HDI}\;+\;\beta }_{2}{\text{URP}\;+\;\beta }_{3}{\text{UNE}\;+\;\beta }_{4}{\text{CRD}\;+\;\beta }_{5}{\text{DK}\;+\;\beta }_{6}{\text{POP}\;+\;u}_{i}{+\;v}_{i}.$$

In the above equation, *α*_0_ is the average case rate or mortality rate for COVID‐19 across all the regions in the world. *U_i_* and *v_i_* are the residual associated at the country and region levels. In all the tests, *p* < 0.05 was assessed statistically significant. The statistical analyses were done with Stata 14 (Stata Corp) software.

The present article is extracted from the research project approved by the Mashhad University of Medical Sciences with Proposal Number 991202. Ethical approval for this study was obtained from Ethics Committee of the Mashhad University of Medical Sciences (The code of Ethics: IR.MUMS.FHMPM.REC.1400.119).

## 3 RESULTS

Table 1 shows socioeconomic characteristics and the outcomes of COVID‐19 in all countries. Until February 12, 2021, the case rate and mortality rate due to the COVID‐19 pandemic were estimated at 5.97, 0.11 per 100,000 person‐day at risk for 184 countries, respectively. The case fatality rate was 2.02%. The rate of COVID‐19 cases varies across countries, ranging from 0.001 (Laos) to 38.87 (Andorra) per 100,000 person‐day. No deaths were reported in 10 countries.

**Table 1:** Socioeconomic characteristics and COVID‐19 outcomes in selected countries

Variable	*N*	Mean ± SD	Median (IQR)	Min (country), max (country)
COVID‐19 cases rate (per 100,000 inhabitants/day at risk)	184	5.97 ± 7.28	2.92 (0.36–10.37)	0.001 (Laos) 38.87 (Andorra)
COVID‐19 mortality rate (per 100,000 inhabitants/day at risk)	184	0.11 ± 0.14	0.03 (0.005–0.18)	0^a^ 0.546 (Slovenia)
Case fatality rate (%)	184	2.02 ± 2.41	1.67 (0.99–2.52)	0^a^ 28.86 (Yemen)
Human Development Index	184	0.721 ± 0.15	0.741 (0.598–0.834)	0.394 (Niger) 0.957 (Norway)
GDP per capita (PPP)	176	20,590.8 ± 20,795.1	13,080.2 (4944. 6–29,869.8)	751.7 (Burundi) 114,481.5 (Luxembourg)
Health expenditure per capita	182	1563.4 ± 1995.6	722.4 (233–2005.3)	30.7 (Congo, DR) 12642.8 (Liberia)
Women population (%)	178	49.8 ± 3.5	50.2 (49.6–50.9)	24.7 (Qatar) 54.4 (Nepal)
Population density per km^2^	182	201.3 ± 627.3	82.5 (35.9–205.9)	2 (Mongolia) 7915.7 (Singapore)
Median age (year)	179	30.1 ± 9.1	29.6 (21.5–38.3)	15.2 (Niger) 48.4 (Japan)
Urban population (%)	184	58.6 ± 22.9	58.9 (40.5–77.7)	13.2 (Papua New Guinea) 100 (Singapore)
Population 65 years or older (%)	178	8.6 ± 6.2	6.1 (3.4–13.9)	1.1 (UAE) 27 (Japan)
Population 70 years or older (%)	178	5.4 ± 4.2	3.5 (2–8.6)	0.5 (UAE) 18.5 (Japan)
Unemployment (%)	178	7.1 ± 5.3	5.3 (3.4–9.7)	0.08 (Qatar) 28.5 (South Africa)
Education index	184	0.657 ± 0.17	0.682 (0.522–0.792)	0.249 (Niger) 0.943 (Germany)
Years of schooling (years)	184	8.7 ± 3.1	8.9 (6.3–11.3)	1.6 (Burkina Faso) 14.2 (Germany)
Cardiovascular disease (per 100,000)	183	6734.4 ± 3316.3	5638.2 (3970.7–9265.9)	2852.4 (Niger) 15,937.5 (Italy)
Diabetes and kidney diseases (per 100,000)	183	12,629.4 ± 5036.1	13,198.4 (8501.8–16,798.9)	4132.5 (Niger) 24,794.2 (Mauritius)
COPD (per 100,000)	183	2385.8 ± 1725	1732.9 (1088.6–2879.9)	516.9 (Fiji) 8200.2 (Demark)
Time since onset in the first confirmed case (day)	184	336.2 ± 46.6	340 (331–350)	21 (Micronesia) 386^b^

Abbreviations: COPD, chronic obstructive pulmonary disease; GDP, gross domestic product; IQR, interquartile range.

^a^ Dominica, Micronesia, Marshal Islands, Vanuatu, Timor–Leste, Saint Kitts and Nevis, Cambodia, Samoa, Laos and Solomon Islands.

^b^ China, the United States, South Korea, Thailand, Japan.

**Figure 1:**
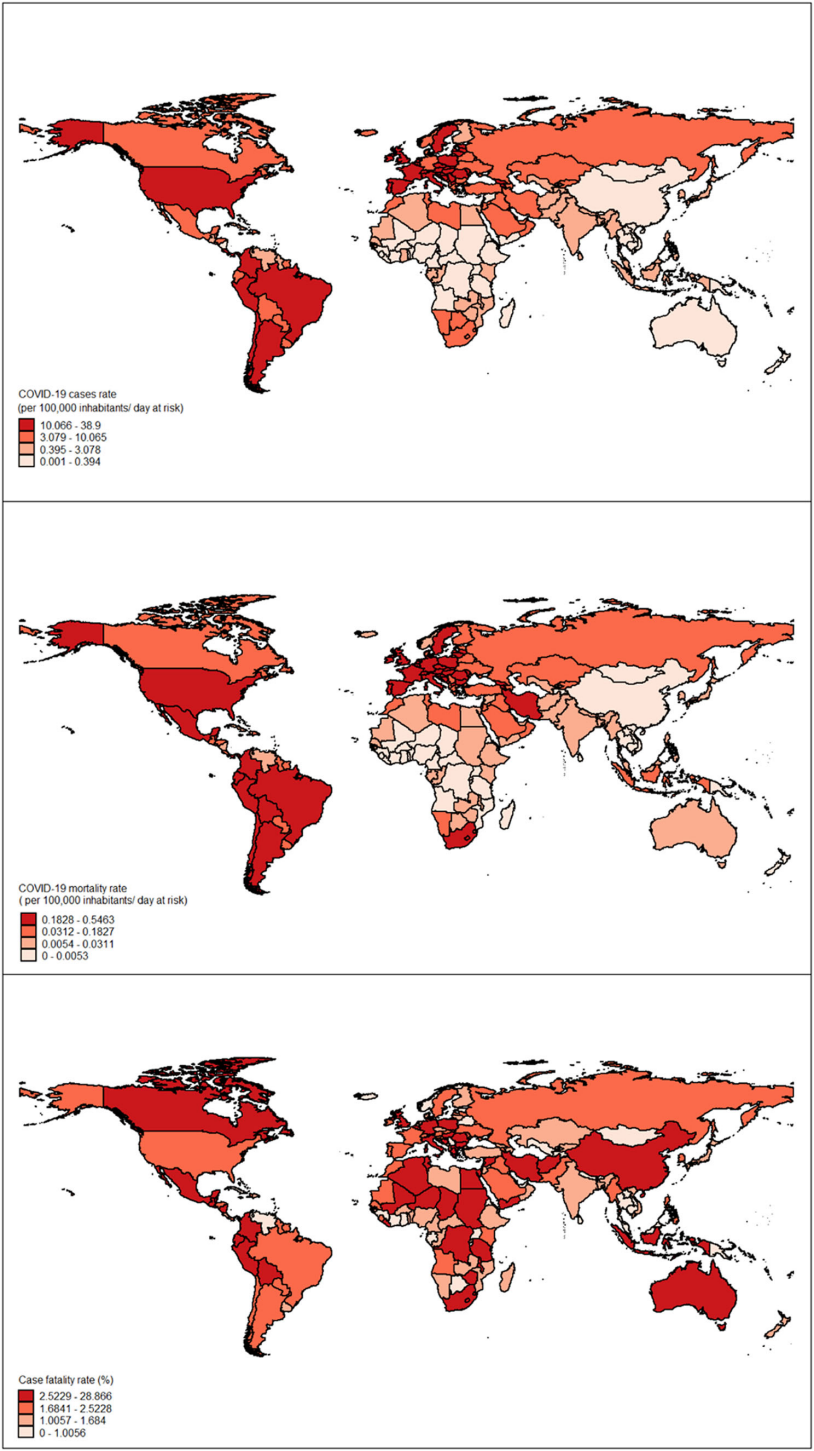
Geographical distribution of the incidence, mortality and case fatality rate of COVID‐19 at country level

As is shown in Figure 1, distinct geographic distribution emerged for COVID‐19‐related variables in the world. The geographic pattern of morbidity and mortality rate was almost similar in the selected countries. For example, the United States of America, some European countries, and Latin American regions had the highest morbidity and mortality rates per 100,000 inhabitants‐day at risk. In contrast, African countries, Australia, and China had the lowest morbidity and mortality rates. However, some counties in Africa and Europe, China, Australia, Canada, and Mexico, are located in high‐risk areas in terms of fatality rate.

Table 2 presents the associations between COVID‐19 outcomes and covariate variables, using correlation coefficient. Among the 15 covariates evaluated, all variables have a significant linear relationship with mortality and morbidity rate, excepting women population and density population. The correlations were stronger (absolute value >0.5) among HDI, urban population, education index, years of schooling, cardiovascular diseases, and age‐related variables. Moreover, there was an association significant linear between case fatality rate by COVID‐19 and women population, the percentage of population 65 years or older, unemployment, population density, cardiovascular disease, and COPD. However, none of these correlations were strong (absolute value >0.25).

**Table 2:** Associations between COVID‐19 variables and socioeconomic characteristics, using correlation coefficient (*r*)

Variable	Case rate per 100,000		Mortality rate per 100,000		Case fatality rate	
	Correlation (*r*)	*T *(*p* Value)	Correlation (*r*)	*T *(*p* Value)	Correlation (*r*)	*T *(*p* Value)
Human development index	0.641	11.28 (<0.001)	0.497	7.74 (<0.001)	0.018	0.25 (0.803)
GDP per capita (PPP)	0.487	7.37 (<0.001)	0.359	5.08 (<0.001)	0.002	0.03 (0.972)
Health expenditure per capita	0.404	5.94 (<0.001)	0.351	5.04 (<0.001)	0.096	1.30 (0.195)
Women population (%)	−0.043	−0.58 (0.56)	0.077	1.03 (0.303)	0.232	3.17 (<0.001)
Population density per km^2^	0.06	0.82 (0.414)	−0.053	−0.72 (0.473)	−0.184	−2.52 (0.012)
Median age (year)	0.592	9.78 (<0.001)	0.52	8.11 (<0.001)	0.097	1.30 (0.195)
Urban population (%)	0.57	9.38 (<0.001)	0.482	7.42 (<0.001)	0.136	1.85 (0.065)
Population 65 year or older (%)	0.515	7.98 (<0.001)	0.492	7.50 (<0.001)	0.164	2.21 (0.028)
Population 70 year or older (%)	0.519	8.06 (<0.001)	0.498	7.63 (<0.001)	0.169	2.28 (0.023)
Unemployment (%)	0.22	3.00 (0.003)	0.254	3.49 (<0.001)	0.187	2.54 (0.012)
Education index	0.606	10.29 (<0.001)	0.478	7.35 (<0.001)	0.032	0.43 (0.666)
Years of schooling (years)	0.595	10.00 (<0.001)	0.468	7.16 (<0.001)	0.03	0.4 (0.689)
Cardiovascular disease (per 100,000)	0.538	8.59 (<0.001)	0.483	7.43 (<0.001)	0.156	2.13 (0.034)
Diabetes and kidney diseases (per 100,000)	0.437	6.54 (<0.001)	0.297	4.19 (<0.001)	−0.08	−1.09 (0.279)
COPD (per 100,000)	0.482	7.40 (<0.001)	0.446	6.70 (<0.001)	0.156	2.13 (0.034)

Abbreviations: COPD, chronic obstructive pulmonary disease; GDP, gross domestic product.

The results of regression models are presented in Table 3. In the linear regression, there was a significant relationship between COVID‐19 cases rate (Log) per 100,000 inhabitants‐day at risk with HDI, percentage of the urban population, unemployment, and the prevalence of cardiovascular disease in 184 countries. Also, the variables of the urban population, unemployment, and cardiovascular diseases were significant predictors for the logarithm of COVID‐19 mortality rate. The results suggest a clear association between HDI and COVID‐19 cases per 100,000 person‐day at risk. The cases of COVID‐19, on average, were 0.384%, 0.578%, and 0.879% per 100,000 inhabitants‐day at risk much higher for medium HDI, high HDI, and very high HDI countries compared with low HDI countries, respectively. In addition, a 1% increase in the urban population was associated with an average of 0.013% and 0.016% increase in the COVID‐19 morbidity and mortality, respectively. The results show that the regression models have a good fit, *R* ^2^ ranging from 0.46 to 0.52, indicating that our models explain between 46% and 52% of the variation in cases and deaths per 100,000 inhabitant‐day in countries.

**Table 3:** Factors associated with COVID‐19 pandemic: Results from the regression models

Variable	(1)	(2)	(3)	(4)
	Log case rate	Log case rate	Log mortality rate	Log mortality rate
	(95% CI)	(95% CI)	(95% CI)	(95% CI)
Fixed effect variables				
HDI				
Low	Ref.	Ref.	Ref.	Ref.
Medium	0.384*	0.539**	0.148	0.461**
	(0.025–0.742)	(0.265–0.814)	(−0.307 to 0.604)	(0.11–0.812)
High	0.578**	0.748**	0.389	0.726**
	(0.095–1.06)	(0.382–1.11)	(−0.225 to 1.00)	(0.257–1.19)
Very high	0.879**	1.065**	0.46	0.825**
	(0.287–1.47)	(0.626–1.50)	(−0.292 to 1.21)	(0.264–1.38)
Urban population (%)	0.013**	0.01**	0.016**	0.009**
	(0.007–0.02)	(0.005–0.014)	(0.008–0.024)	(0.003–0.015)
Unemployment (%)	0.035**	0.018*	0.048**	0.023*
	(0.015–0.055)	(0.004–0.033)	(0.022–0.073)	(0.005–0.042)
Cardiovascular disease	0.0001**	0.00004	0.0001*	0.00005
	(0.00002–0.0001)	(−0.00001 to 0.0001)	(0.00002–0.0002)	(−0.00002 to 0.0001)
Diabetes and kidney diseases	−0.00002	−0.00001	−0.00005	−0.00003
	(−0.00006 to 0.00001)	(−0.00004 to 0.00001)	(−0.0001 to 0.000001)	(−0.00008 to 0.000001)
Population 65 years or older (%)	−0.019	−0.018	0.004	0.023
Constant	(−0.065 to 0.027)	(−0.056 to 0.019)	(−0.055 to 0.063)	(−0.025 to 0.071)
	−1.606**	−1.32**	−3.587**	−3.173**
	(−2.05 to 1.15)	(−1.90 to 0.74)	(−4.16 to 3.01)	(−3.91 to 2.42)
Random effect variable	‐		‐	
Variance Level 1		0.311		0.52
		(0.096–1.009)		(0.16–169)
Variance Level 2 (WHO region)		0.216		0.354
		(0.175–0.268)		(0.286–0.439)
Model summary				
Number of observations	176	176	176	176
Number of groups	‐	6	‐	6
*R* ^2^	*R* ^2^: 0.52	‐	*R* ^2^: 0.46	‐
*R* ^2^adjusted	*R* ^2^adjusted: 0.50	‐	*R* ^2^adjusted: 0.43	‐
*F*/Likelihood ratio (LR)	23.52	LR: 92.65	17.8	LR: 90.68

Abbreviations: HDI, human development index; WHO, World Health Organization.

* *p* < 0.05

** *p* < 0.01.

In the multilevel analysis, there was a significant association at the *p*‐value of 5% level between outcomes of COVID‐19, that is, morbidity and mortality, with HDI, percentage of the urban population, and unemployment. Furthermore, the multilevel analysis displayed that the variances are varied between Level 1 (country level) and Level 2 (WHO regions), meaning that the geographic distribution represented a proportion of the changes in the COVID‐19 outcomes. For example, Model 2 estimated the variance of 0.311 across countries and 0.216 over regions, suggesting that approximately 40% (0.216/0.527) of changes in case rate are due to the geographic environment.

## 4 DISCUSSION

Our ecologic study in 184 countries indicated that the socioeconomic status and geographic distribution are essential contributors to the COVID‐19 pandemic. In the assessed variables, the HDI, urban population, unemployment, and the prevalence of cardiovascular diseases were significantly associated with morbidity and mortality rate by COVID‐19. In addition, the results are shown that the COVID‐19 outcomes are varied based on the geographical location of countries.

The results demonstrate a strong association between the HDI and COVID‐19 outcomes at the global level. The countries with higher HDI have higher COVID‐19 morbidity and mortality rates compared with low HDI countries. In other words, the case and death rate of COVID‐19 per 100,000 inhabitant‐day at risk were more increased on average in developed countries. According to the regression models, the COVID‐19 morbidity was estimated between 0.87% and 1.06% per 100,000 person‐day, higher for high HDI countries compared with low HDI countries, on average. Additionally, the death rate by COVID‐19 was approximately calculated at 0.82% more for high HDI countries versus low HDI countries. We can consider some reasons why morbidity and mortality are higher in more developed countries. First, the COVID‐19 pandemic started in China early and then spread to the developed countries such as the United States and the European Union, eventually reaching the African countries. Therefore, the developed countries are likely to be in the final stage of the virus compared to other countries. Second, another reason could be that the countries with high HDI usually have better records than low HDI countries. Therefore, the case and death number due to COVID‐19 may be underestimated in countries with a lower HDI. Also, the latent cases are high in COVID‐19, especially in developing countries, because these countries have less access to diagnostic tests. Third, socioeconomic factors affect the COVID‐19 outcomes as well as health status. These variables could have been a notable effect on morbidity and mortality; sometimes the simultaneous effects of some variables could aggregate disease outcomes or vice versa. However, studies have shown that the COVID‐19 outcomes are significantly associate with the prevalence of cardiovascular diseases, diabetes, and kidney and COPD. In this respect, Azarpazhooh et al.^16^ assessed the COVID‐19 pandemic and burden of non‐communicable diseases, using an ecological study in 184 countries. They showed a positive correlation between noncommunicable disease DALYs with case and death rate on COVID‐19. Another ecological study by Rodriguez‐Villamizar et al.^17^ reported that the percentage of population 65 years or older, poverty index, and the prevalence of hypertension above 6% are the main determinants of the death rate by COVID‐19. As mentioned, the prevalence of cardiovascular disease and the elderly population is higher in developed countries, so it can be expected that a proportion of the higher COVID‐19 mortality rate could be the results of these variables. Our regression models revealed that the prevalence of the cardiovascular disease is a significant predictor of COVID‐19 morbidity and mortality.

Numerous studies have investigated the role of the socioeconomic variable on the health outcome via an ecological study approach.^18^, ^19^, ^20^, ^21^ In this regard, socioeconomic status was assessed as a predictor of the COVID‐19 outcomes (mortality and morbidity) in earlier studies,^1^, ^16^, ^17^, ^22^ none of these studies examined the geographical location of countries. We indicated that the geographical distribution of the country location is a significant variable on the case and death rate per 100,000 person‐day at risk by COVID‐19. Using multilevel analysis, we found that nearly 40% of the case rates (0.216/0.527) and death rates (0.354/0.874) changes are associated with the geographic environment. In other words, the individual characteristics within countries are determinants for COVID‐19 outcomes but adding WHO regions as a second‐level variable could be shown as a part of changes between countries. Furthermore, in the geospatial analyses, particular patterns were derived from COVID‐19 outcomes, indicating that countries responded to the disease differently. For example, although having a high mortality and morbidity rate, some countries in the European Union had relatively lower case fatality rates. In contrast, countries like China, Australia, and African countries had low cases and deaths rates but high case fatality rates.

There are limitations to these findings. We have done an ecological study at the country and region level. Using the results of ecological studies should be done with caution about the individual level, which is an unavoidable limitation of these studies. Additionally, the governments have played an essential role in controlling the COVID‐19 pandemic (covering the expenses for PCR testing and treatment, collecting and reporting information, preparedness, and deciding for lockdown). The reported data associated with COVID‐19 morbidity can be affected by the accessibility and availability of testing for the disease in a country or region. Therefore, the calculated rates may be underestimated in some countries, not because those countries have a lower risk of disease but have less access to diagnostic tests. This problem is more likely to happen in low‐income countries. Of course, we used person‐day at the time risk as a denominator in estimating rates, leading to more realistic estimates.

Nevertheless, ecological studies are the foundation to designing hypotheses at the individual level, so it can be hypothesized that socioeconomic factors and geographical environment can directly or indirectly affect the COVID‐19 outcomes. Addressing these factors can improve health outcomes. These hypotheses could be a subject for upcoming studies at an individual level.

## 5 CONCLUSION

The study suggests that in addition to the socioeconomic status affects the COVID‐19 outcomes at the country level, countries' geographical location makes a part of changes in outcomes of diseases. Therefore, health policymakers could overcome morbidity and mortality in COVID‐19 by controlling the socioeconomics factors.

## AUTHOR CONTRIBUTIONS

**Ahmad Faramarzi**: Conceptualization; data curation; formal analysis; methodology; visualization; writing—original draft; writing—review & editing. **Javad Javan‐Noughabi**: Conceptualization; formal analysis; methodology; project administration; software; supervision; writing—original draft; writing—review & editing. **Sayed Ali Mousavi**: Data curation; writing—original draft; writing—review & editing. **Farshad Bahrami Asl**: Data curation; investigation; writing—review & editing. **Hamidreza Shabanikiya**: Investigation; validation; writing—review & editing.

## CONFLICTS OF INTEREST

The authors declare no conflicts of interest.

## TRANSPARENCY STATEMENT

The corresponding author confirm that “manuscript is an honest, accurate, and transparent account of the study being reported; that no important aspects of the study have been omitted; and that any discrepancies from the study as planned (and, if relevant, registered) have been explained”.

## ETHICS STATEMENT

The present article is extracted from the research project approved by the Mashhad University of Medical Sciences with Proposal Number 991202. Ethical approval for this study was obtained from Ethics Committee of the Mashhad University of Medical Sciences (The code of Ethics: IR. MUMS. FHMPM. REC.1400.119).

## Data Availability

The data that support the findings of this study are available from the corresponding author upon reasonable request.
